# First clinical experience with the novel Varipulse Pro platform for ablation of atrial fibrillation

**DOI:** 10.1093/ehjcr/ytag248

**Published:** 2026-04-03

**Authors:** Stjepan Jurisic, Nadica Trajkovska, Philipp Sommer, Christian Sohns

**Affiliations:** Department of Electrophysiology, Heart and Diabetes Center NRW, Ruhr University Bochum, Georgstr. 11, Bad Oeynhausen 32545, Germany; Department of Cardiology, University Heart Center, University Hospital Zurich, Raemistrasse 100, Zurich 8091, Switzerland; Department of Electrophysiology, Heart and Diabetes Center NRW, Ruhr University Bochum, Georgstr. 11, Bad Oeynhausen 32545, Germany; Department of Electrophysiology, Heart and Diabetes Center NRW, Ruhr University Bochum, Georgstr. 11, Bad Oeynhausen 32545, Germany; Department of Electrophysiology, Heart and Diabetes Center NRW, Ruhr University Bochum, Georgstr. 11, Bad Oeynhausen 32545, Germany

## Case description

**Figure 1:**
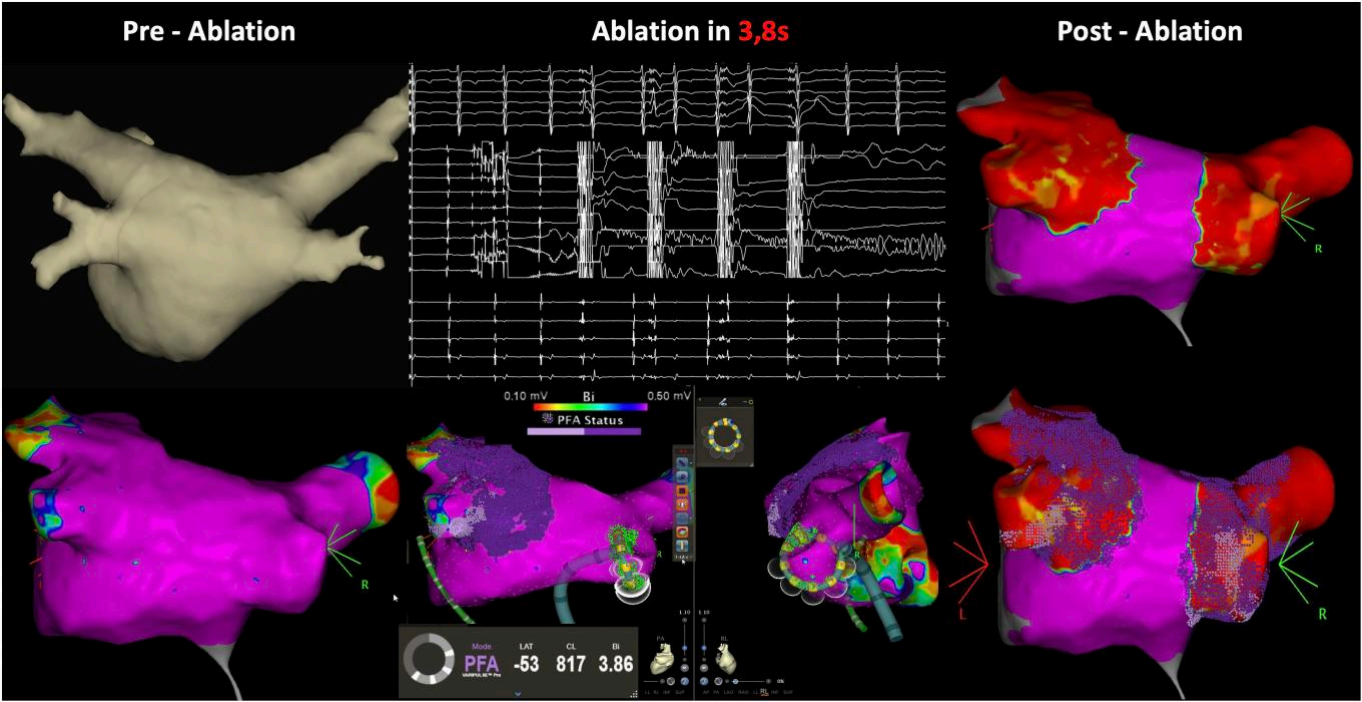
Pre-ablation imaging (left) includes computed tomography-based anatomical reconstruction and 3D electroanatomical voltage mapping of the left atrium and pulmonary veins. Intraprocedural recordings (centre) show intracardiac electrograms during pulsed field ablation (PFA), including tissue proximity indicator (TPI) guidance and grid visualization. Post-ablation mapping (right) illustrates lesion formation, with corresponding voltage reduction and ablation grids.

A 64-year-old patient with a 6-month history of symptomatic atrial fibrillation (AF) was scheduled for index AF ablation. The procedure was performed using the novel Varipulse Pro platform (Varipulse, JNJ MedTech Inc., Irvine, CA, USA). Preprocedural computed tomography imaging revealed two right-sided and two left-sided pulmonary veins (*Figure 1*, upper left panel). The procedure was performed under unconscious sedation in accordance with our institutional protocol.^1^

Voltage mapping (CARTO™, JNJ MedTech Inc., Irvine, CA, USA) demonstrated no low-voltage areas in the left atrium (LA). After achieving an activated clotting time of >300 s, pulmonary vein isolation (PVI) was performed using the circular variable-loop catheter, incorporating the novel ultra-fast Pro protocol. Ablation was guided by the tissue proximity indicator (TPI), which provides real-time feedback on catheter–tissue contact to optimize lesion formation.^2^

Each pulmonary vein was isolated with four ablation trains at slightly different positions, each consisting of four pulsed field (PF) impulses. Each train was delivered within 3.8 s, resulting in a markedly reduced total energy delivery time.^3^ Electrogram recordings demonstrated immediate elimination of pulmonary vein potentials following the first PF impulse (*Figure 1*, upper middle panel).

Acute isolation was achieved in all pulmonary veins, with a total procedure time (skin-to-skin) of 21 min. No significant coughing or patient movement was observed during PF applications. Repeat voltage mapping of the LA confirmed a very high overlap between TPI-guided PF applications, indicating effective lesion formation (*Figure 1*, right panel).

This case report demonstrates the effectiveness of the novel Varipulse Pro platform for pulmonary vein isolation in a clinical setting. The combination of a 3.8-s application duration and precise TPI guidance enables rapid procedures with effective pulmonary vein isolation.

## Data Availability

The original data are available from the authors upon reasonable request.

## Notes

**Statement of Consent:** Written informed consent was obtained from the patient for publication of this case and all accompanying images.
